# Adherence to Beers Criteria in Geriatrics: A Retrospective Study in a Saudi Teaching Hospital

**DOI:** 10.3390/geriatrics5040097

**Published:** 2020-11-21

**Authors:** Samah Alshehri, Mohannad Alshibani, Ghaydaa Magboul, Albandari Albandar, Roaa Nasser, Roaya M. Yaqoub, Jumana Alzuhayri, Ahmed Aljabri

**Affiliations:** 1 Department of Pharmacy Practice, Faculty of Pharmacy, King Abdulaziz Univeristy, Jeddah 21589, Saudi Arabia; malshibani@kau.edu.sa (M.A.); amaljabri@kau.edu.sa (A.A.); 2 Faculty of Pharmacy, University of Arizona, Tucson, AZ 85721, USA; 3 Faculty of Medicine, King Abdulaziz Univeristy, Jeddah 21589, Saudi Arabia; Ghayda950@gmail.com (G.M.); Albanderi292@gmail.com (A.A.); Roaanasser@gmail.com (R.N.); Roromajedx212@gmail.com (R.M.Y.); 4 Faculty of Science, King Abdulaziz Univeristy, Jeddah 21589, Saudi Arabia; J.alzuhayri@gmail.com; 5 Department of Pharmacy Practice, Faculty of Pharmacy, University of Tabuk, Tabuk 71491, Saudi Arabia

**Keywords:** adherence, Beers criteria, geriatrics, potential inappropriate medications

## Abstract

Background: The aging process makes geriatric populations more prone to various chronic diseases. Such diseases require older patients to be on more medications than any other age group and make them more susceptible to adverse drug events related to potentially inappropriate medications (PIMs). Aim: To identify the prevalence of potentially inappropriate medications among older people and explore the most commonly prescribed PIMs in hospitalized patients. Design and Setting: A retrospective study conducted in a large tertiary hospital among patients hospitalized in a 4 year period from January 2015 to December 2018. Methods: The 2019 Beers Criteria were used to assess PIMs in all inpatient prescribed medications focusing on the first class (i.e., drug/drug class to be avoided in older adults). Results: The mean age was 75.17 ± 7.66 years. A total of 684 (80.6%) patients were prescribed at least one medication listed in the first-class category of the 2019 Beers Criteria. Top five drugs were proton pump inhibitors (40.3%), nonsteroidal anti-inflammatory drugs (10.2%), metoclopramide (9.3%), benzodiazepines (8.4%), and insulin (5.4%). Conclusions: The prevalence of PIMs is high among older patients admitted to the hospital. More efforts are needed to investigate the potential reasons and develop action plans to improve concordance to Beers Criteria among healthcare providers.

## 1. Introduction

The aging process makes geriatric populations more prone to various chronic diseases [1]. Such diseases require older patients to be on more medications than any other age group and make them more susceptible to adverse drug events related to potentially inappropriate medications (PIMs) [2,3]. PIMs are defined as medications for which the risks for older people outweigh the benefits [4]. In 1991, the Beers Criteria were established to prevent adverse drug effects due to PIMs in geriatric patients. The Beers Criteria have had several updates, and the most recent version was published in 2019 in association with the American Geriatric Society [5].

Several studies have addressed the prevalence of PIMs in geriatric patients globally; in Canada and the United States, the prevalence was from 14% to 37%, whereas in Europe it was from 23% to 43% [6]. A retrospective study in Indonesia in 2014 reported a PIMs prevalence of 52.2% [7]. Moreover, studies with lower rates were reported in South Africa, Korea, and Nigeria, with prevalences of 13.8%, 27.6%, and 32.1%, respectively [8,9]. Higher rates of 40.39%, 45.2%, and 53.5% were reported in New Zealand, Lebanon, and China, respectively [10,11,12].

A 2018 study in Saudi Arabia using the 2012 Beers Criteria revealed that 80% of patients were using at least one listed PIM. The study showed high PIM rates of 72.6%, 59.2%, and 37.7% for the first, second, and third class of Beers Criteria, respectively. In addition, the study noted that insulin and nonsteroidal anti-inflammatory drugs (NSAIDs) were the most commonly prescribed PIMs [13].

The United Nations expects that the population of Saudi older adults will continue to increase through to 2050 [14]. With this predicted expansion, scholars have to bring older adults’ healthcare into focus through assessing prescription medication quality and screening geriatrics for adherence to Beers Criteria. A study conducted in Jeddah, Saudi Arabia to examine the characteristics of hospitalized patient found that older adults accounted for 16.8% of the population admitted to the hospital [15]. There were no data to examine the pattern of medication prescribing in older adults in Saudi Arabia.

The aim of this study was to identify the prevalence of PIMs among in-patient old people admitted to a tertiary hospital in Jeddah, Saudi Arabia, and how prevalence is distributed per age group, and to explore the most commonly prescribed PIMs.

## 2. Materials and Methods

This retrospective study was conducted in a tertiary hospital in Jeddah in the inpatient setting among patients hospitalized in a 4 year period from January 2015 to December 2018 to identify the prevalence of PIMs among older adults as well as the most commonly prescribed PIMs in the inpatient setting. The hospital provides various medical services in the western area and has a capacity of 1002 beds.

The target sample of this study included patients 65 years or older who were admitted during the last four years. Patients with liver cirrhosis or chronic kidney disease stage 4 or 5, on dialysis, admitted to the oncology department or intensive care unit, or with missing profiles were excluded. The hospital’s biomedical ethics unit approved this study.

The data for eligible patients for the prior four years were obtained from the electronic medical record, extracted into a data collection sheet, and retrospectively reviewed for PIMs. Patients were grouped according to their age as follows: 65–75, 76–85, and >85 years. The sheet included the patients’ medication profiles and demographic data, including age and sex.

The data collectors were introduced to Beers Criteria and PIMs, and then the data were entered and saved in protected devices. The 2019 Beers Criteria were used to assess for PIMs in all prescribed medications. PIMs are organized into three categories according to Beers Criteria: (a) first class (i.e., medications to be avoided in geriatrics regardless of their conditions), (b) second class (i.e., medications to be avoided with certain syndromes or diseases), and (c) third class (i.e., medications to be used with caution). However, the focus of our study was on the first class only.

Data analysis was performed using SPSS version 25. Descriptive statistics were used for univariates. Categorical and nominal variables were presented as percentages while means and standard deviations (SDs) were used for continuous variables.

## 3. Results

**Figure 1:**
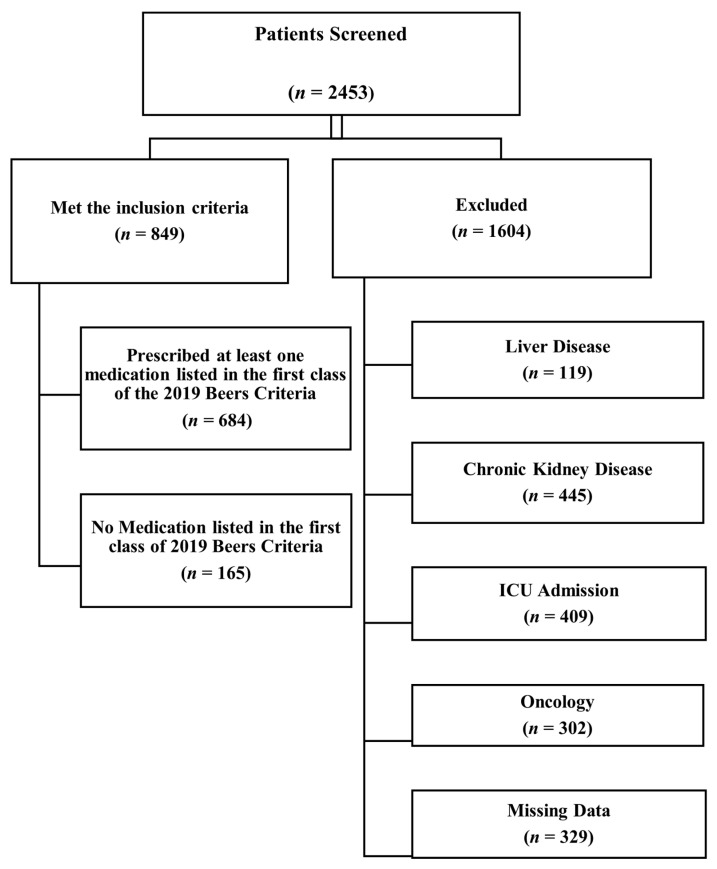
Flow of patients throughout the study.

A total of 2453 patients were screened, and 849 patients met the inclusion criteria Figure 1. Overall, there were 438 (51.6%) males and 465 Saudis (54.8%) in our sample. Other common nationalities included Yemenis 182 (21.4%), Palestinians 29 (3.4%), Syrians 21 (2.5%), and Sudanese 21 (2.5%). The mean age was 75.17 ± 7.66 years. Stratifying the patients according to their age, 57.3% were between 65 and 75 years, 32.3% were between 76 and 85 years, and 10.4% of the patients were >85 years. Table 1 shows the baseline characteristics of the population.

A total of 684 (80.6%) of the patients were prescribed at least one medication listed in the first class of the 2019 Beers Criteria (1839 prescriptions/orders). There was no deference in the age between the adherent and non-adherent group 74.52 ± 7.6 and 75.31 ± 7.7 years; *p* = 0.94. There was no gender difference in adherence rate: 355 (81.1%) in males compared to 329 (80.0%) in females; *p* = 0.568.

Of the 34 PIMs identified as shown in Table 2, the top five prescribed medications or medication classes were proton pump inhibitors (PPIs) 741 (40.3%) prescriptions/orders, NSAIDs 187 (10.2%) prescriptions/orders, metoclopramide 171 (9.3%) prescriptions/orders, benzodiazepines 155 (8.4%) prescriptions/orders, and insulin 99 (5.4%) prescriptions/orders.

## 4. Discussion

### 4.1. Summary

The aim of our study was to identify the prevalence of PPIs among geriatric patients in an inpatient setting. We found that 80.6% of patients were prescribed at least one medication listed in the first class of the 2019 Beers Criteria. The top five drugs were PPIs (40.3%), NSAIDs (10.2%), metoclopramide (9.3%), benzodiazepines (8.4%), and insulin sliding scale (5.4%).

### 4.2. Strengths and Limitations

To the best of our knowledge, there are no published studies that evaluate the prevalence of PIMs in accordance with the updated version of the 2019 Beers Criteria in Saudi Arabia. This study identified the most common PIMs among older patients admitted to one of the largest tertiary hospitals in the western region of Saudi Arabia, with the intent to encourage prescribers to use the 2019 Beers Criteria.

There were multiple limitations to the present study. First, the sample was collected retrospectively from in-patients only, which does not represent the geriatric population. Second, this study included the first class of Beers Criteria exclusively, in which not all medications listed in that class were available in our center formulary. Additionally, the indication and duration of therapy of the prescribed medications were not evaluated.

### 4.3. Comparison with Existing Literature

Although several drugs were removed from the new Beers Criteria, the prevalence of PIMs was higher in the current study compared to that in two previous studies conducted in Saudi Arabia that relied on older versions of the Beers Criteria. The first study found the prevalence to be 72.6% in a 135 population-based study conducted in Jeddah [13], whereas the other study reported a prevalence of 61% among 400 patients in Riyadh [16].

Several potential reasons contribute to the high prevalence of PIMs. One of the potential reasons is that many prescribers comfortably rely on the same medication for years and they are suspicious of substitutes. Prescribers must take into consideration that many of the medications are not universally appropriate but rather depend upon a patient’s circumstances [17]. In particular, prescribers should be aware of the rationale of including the medication in the Beers Criteria [17]. Moreover, health systems in some institutions lack the privilege of flagging medication listed in Beers Criteria for extra caution. Furthermore, increased PIMs among geriatrics can be attributed to the use of multiple medications, also known as polypharmacy, which has increased in geriatrics in the recent years [18,19].

According to our results, the most frequently prescribed PIM was PPIs with a rate of 40.3%. This result was consistent with a Chinese retrospective cross-sectional study that was conducted in 2017 in which the PPI use rate was 41.9% [12]. Histamine-2 receptor antagonists can be safely used as alternative medication to PPIs, but the Beers Criteria recommend against its use in patients with delirium due to risk of worsening their conditions [5]. This exception may create potential confusion to avoid using histamine-2 receptor antagonists for all old people, which highlights the role of pharmacists in increasing the awareness of its potential use for old people as an alternative to PPIs in most cases. Other nonpharmacological therapy such as exercise and psychological therapy can be suggested; however, patients may not be willing to consider it [1,20].

In addition, the second most common PIM implicated in our study was NSAIDs with a rate of 10.2%. Similarly, a rate of 10.17% was reported in one of the hospitals in Jeddah because it was mostly prescribed for chronic kidney disease and heart failure patients [13]. Because NSAID are widely used, adverse effects have to be addressed, including increased risk of stroke, cardiovascular death, gastrointestinal hemorrhage, and peptic ulcer disease, especially in high-risk groups that include patients older than 75 taking oral or parenteral corticosteroids, anticoagulants, or antiplatelet agents [21]. According to a literature review published in 2015, topical NSAIDs, lidocaine patch, topical capsaicin cream, and acetaminophen are potential alternatives to oral NSAID therapy for chronic pain due to the ease of use and lower risk of adverse effects [22].

The third most frequently prescribed PIM was metoclopramide (9.3%). In a study conducted in India, metoclopramide was the number one drug on the list of PIMs with a remarkably high rate of 54.3% [23]. Metoclopramide has a negative effect on health because it can possibly cause extrapyramidal adverse reactions. In addition, the long-term use of metoclopramide can adversely cause persistent tardive dyskinesia [24]. Overall, it should be avoided among geriatric patients and cautiously used for a duration <12 weeks in patients who suffer from gastroparesis, with some exceptions in rare cases. Ondansetron can be used as an alternative medication [5].

The fourth most frequently prescribed PIM identified in this study was attributable to benzodiazepines (8.4%), including diazepam (6.0%) and lorazepam (2.4%). In China, a study that compared the use of benzodiazepines reported a change in the rank of benzodiazepines from the most frequent PIM in 2012 to the second most frequent in 2015 after PPIs [12]. According to the American Geriatric Society, benzodiazepines are known to increase the risk of cognitive impairment, delirium, and fractures. It should be noted that older adults have a lower rate of metabolism of a long-acting agent [24]. Using serotonin–norepinephrine reuptake inhibitors as alternative medication to benzodiazepines is recommended, except for patients with a history of high risk of falls. In addition, buspirone might be used as an alternative for a patient who has anxiety [5]. Furthermore, previous studies have shown that central nervous system medications such as benzodiazepines had minimal effectiveness on sleep in geriatric patients. It is worth mentioning that some nonpharmacological options exist to treat insomnia, including a combination of sleep hygiene and behavioral intervention [22].

Lastly, insulin sliding scale was the fifth most commonly prescribed PIM (5.4%). Insulin has been revised in the 2019 Beers Criteria to minimize the confusion about inappropriate insulin regimens. The criteria advice is to avoid using insulin sliding scale with short- or rapid-acting insulin without concurrent use of basal or long-acting insulin to minimize the risk of hypoglycemia [5]. A study conducted in the same city found that insulin sliding scale is the most reported PIM in their institute, affecting 56 of 135 (41.5%) patients [13].

### 4.4. Implications for Research and/or Practice

Our data suggest that the prevalence of PIMs is high among older patients admitted to our hospital. It is recommended to routinely practice applying new strategies for improving the dispensing of any medications to the older adults and hospitalized older adults. Strategies such as medication reconciliation, electronic system messages to alert the physician when they order PIMs in older adults, and education sessions about the alternative safe medications for the top PIMs identified should be implemented. More efforts are needed to investigate the potential causes behind that high prevalence and to develop action plans to improve concordance to Beers Criteria among healthcare providers in inpatient settings.

It is worth mentioning that the Beers Criteria is a great quality-improving tool in clinical settings. The criteria are designed to support, instead of replacing, good clinical judgment. However sometimes it may be misinterpreted and implemented in ways that cause unwanted harm. The criteria aid in identifying medications whereby the harm exceeds the benefits in many geriatric patients, especially in comparison to pharmacologic and nonpharmacologic options. Nevertheless, in some circumstances the use of medications included in the criteria can be appropriate [17].

## 5. Conclusion

The results of the current study showed that the prevalence of PIMs is high among elderly patients admitted to the hospital. The findings highlight the need for more efforts to investigate potential reasons and action plans to improve concordance to Beers Criteria among healthcare providers.

### 5.1. Practice Impact Statement

This study identified the most common potentially inappropriate medications (PIMs) among older patients admitted to one of the largest tertiary hospitals in the western region of Saudi Arabia, with the intent to encourage prescribers to use the 2019 Beers Criteria. This is the first study that identify the frequency of PIM use in inpatients setting using the 2019 Beers Criteria in Saudi Arabia.

### 5.2. How This Fit In

The most recent version of the Beers Criteria was published in 2019 in association with the American Geriatric Society. Numerous studies have addressed the prevalence of PIMs in geriatric patients globally. To the best of our knowledge, there are no published studies that evaluate the prevalence of PIMs in accordance with the recent criteria in Saudi Arabia.

## Figures and Tables

**Table 1:** General characteristics of the patients included in the study.

Characteristics *N* (%)	All Ages 849	65–75 years 487 (57.3%)	76–85 years 274 (32.3%)	>85 years 88 (10.4%)
Age Mean ± SD	75.17 ± 7.66	69.74 ± 3.05	79.76 ± 2.74	90.90 ± 3.88
Nationality Saudi, *n* (%)	465 (54.8)	260 (53.4)	151 (55.1)	54 (61.4)
Gender Male, *n* (%)	438 (51.6)	248 (50.9)	143 (52.2)	47 (53.4)
Beers Criteria Medication Identified	684 (80.6%)	387 (79.5%)	226 (82.5%)	71 (80.7%)

SD: standard deviation; *n*: number of patients; %: percentage.

**Table 2:** The most commonly encountered potentially inappropriate medication (PIMs) as per the 2019 Beers Criteria.

Medication	All Ages 849 (100%)	65–75 487 (57.3%)	76–85 274 (32.3%)	>85 88 (10.4%)
PPI	741 (40.3%)	435 (23.7%)	251 (13.7%)	55 (3.0%)
NSAID	187 (10.2%)	121(6.6%)	58 (3.2%)	8 (0.4%)
Metoclopramide	171 (9.3%)	100 (5.4%)	55 (3.0%)	16 (0.9%)
Benzodiazepines	155 (8.4%)	85 (4.6%)	57 (3.1%)	13 (0.7%)
Insulin	99 (5.4%)	67 (3.6%)	25 (1.4%)	7 (0.4%)

%: percentage; PPIs: proton pump inhibitors (omeprazole and pantoprazole); NSAIDs: nonsteroidal anti-inflammatory drugs (diclofenac and ibuprofen); Benzodiazepines: (diazepam and lorazepam); Insulin: insulin sliding scale.
